# Supplementary material for: Polyphenols as Potential Protectors against Radiation-Induced Adverse Effects in Patients with Thoracic Cancer
Source: Cancers (Basel). 2023 Apr 22;15(9):2412. doi: 10.3390/cancers15092412 (PMC10177176; doi:10.3390/cancers15092412)
Supplement: Supplementary file 1 [file cancers-15-02412-s001.zip › cancers-2325298-supplementary.pdf]

Supplementary material

Table S1. Radioprotective effects of polyphenols in other healthy tissues.

Compound	Effect	In vitro studies	In vivo studies	Clinical studies
**Soy isoflavones**	**↓ DNA damage**	Liver [107]	Liver [108]; intestine [109]	-
	**Anti-oxidant**	-	Testicle [110]	-
	**Anti-inflammatory**	Prostate [111]	-	-
	**Other**	-	Blood cells [112,113]	Urinary, intestinal and sexual function in prostate cancer patients (NCT00243048) [114]
**EGCG**	**↓ DNA damage**	Intestine [115]; spleen [116]; skin [117]	Intestine [115]	-
	**Anti-oxidant**	Skin [117]; Intestine [115]; spleen [116]; salivary glands [118]	Brain [119]; intestine [115]; liver [120]	-
	**Anti-inflammatory**	-	Brain [119]	-
	**Other**	Retina [121]	Intestine [115]; testicle [122]; blood cells [123]	Mucositis in head and neck cancer patients (NCT01481818) [124]; radiodermatitis in breast cancer patients (NCT01481818) [125,126]
**Silibinin**	**↓ DNA damage**	Kidney [127,128]	Lymphocytes [129]; sperm [130]	
	**Anti-oxidant**	Kidney [127,128]	Liver [131]	-
	**Anti-inflammatory**	-	Intestine [132]; skin [133]	-
	**Other**	Skin [134]	Sperm [130,135]	Mucositis in head and neck cancer patients (IRCT2015050622132N1) [136]; radiodermatitis in breast cancer patients [42], (IRCT2016110730760N1) [137]
**Quercetin**	**↓ DNA damage**	Lymphocytes [138,139]	Oral mucosa [140]; kidney, bladder [141]	-
	**Anti-oxidant**	Neutrophils [142]; skin [143]	Brain [144]; erythrocytes [145]; kidney, bladder [141]; liver [146,147]; oral mucosa [140]; salivary glands [148]; spleen [149]	-
	**Anti-inflammatory**	Lymphocytes [138]; neutrophils [142]	Brain [144]; kidney, bladder [141]; intestine [150]; liver [151]; oral	-

Compound	Effect	In vitro studies	In vivo studies	Clinical studies
			mucosa [140]; skin [150,152]; spleen [149]	
CAPE	Anti-oxidant	Liver [153,154]	Intestine [155]; liver [156,157]	-
	Anti-inflammatory	Liver [153]; macrophage [158]	Intestine [159,160]; liver [155,156]	-
Curcumin	↓ DNA damage	Lymphocytes [161]	-	-
	Anti-oxidant	Lymphocytes [161]; liver [162,163]; skin [164]	Brain [165]; eye [166]; liver [167]; skin [168]	-
	Anti-inflammatory	Endothelium [169]	Liver [167]; skin [170,171]	-
	Other	-	Intestine [172]; oral mucosa [173]	Radiodermatitis in breast cancer patients (NCT01042938) [43], (NCT01246973) [174], (NCT02536632) [175], (IRCT20200513 047427N1) [176]
Thymol	↓ DNA damage	-	Blood cells [177]	-
	Anti-oxidant	-	Liver [177]; ovary [178]; salivary glands [179]	-
	Other	-	Intestine [177]; salivary glands [180]	-
Zingerone	↓ DNA damage	Lymphocytes [181]	-	-
	Anti-oxidant	Lymphocytes [181]	Liver [182,183]	-
	Other	-	Intestine [184]	-
Resveratrol	↓ DNA damage	Lymphocytes [185]; kidney [186,187]	Bone marrow [188]; lymphocytes [189]	-
	Anti-oxidant	Skin [190]	Bone marrow [191]; brain [192]; intestine [193,194]	-
	Other	Intestine [195]	Brain [196]; intestine [197]; salivary glands [198]	Radiodermatitis in breast cancer patients [199]
SDG	Anti-oxidant	-	Liver [200]	-
